# The Expression Pattern of EVA1C, a Novel Slit Receptor, Is Consistent with an Axon Guidance Role in the Mouse Nervous System

**DOI:** 10.1371/journal.pone.0074115

**Published:** 2013-09-09

**Authors:** Gregory James, Simon R. Foster, Brian Key, Annemiek Beverdam

**Affiliations:** School of Biomedical Science, University of Queensland, Brisbane, Australia; Trinity College Dublin, Ireland

## Abstract

The Slit/Robo axon guidance families play a vital role in the formation of neural circuitry within select regions of the developing mouse nervous system. Typically Slits signal through the Robo receptors, however they also have Robo-independent functions. The novel Slit receptor Eva-1, recently discovered in *C. elegans*, and the human orthologue of which is located in the Down syndrome critical region on chromosome 21, could account for some of these Robo independent functions as well as provide selectivity to Robo-mediated axon responses to Slit. Here we investigate the expression of the mammalian orthologue EVA1C in regions of the developing mouse nervous system which have been shown to exhibit Robo-dependent and -independent responses to Slit. We report that EVA1C is expressed by axons contributing to commissures, tracts and nerve pathways of the developing spinal cord and forebrain. Furthermore it is expressed by axons that display both Robo-dependent and -independent functions of Slit, supporting a role for EVA1C in Slit/Robo mediated neural circuit formation in the developing nervous system.

## Introduction

The Slit/Robo families are chemorepulsive cues that were first identified in *Drosophila*, where they are most well-known for regulation of midline crossing by commissural axons [1]. The axon guidance function of Slit/Robo signaling is highly conserved in mice and humans and appears to be associated with some neurological disorders. The cortical innervation defects that occur in the absence of Slit/Robo signaling in mice resemble defects present in mouse models of Down syndrome [2], [3]. Moreover, defects in Slit/Robo signaling disrupt commissural axon crossing in the hindbrain in horizontal gaze palsy with progressive scoliosis [4], [5]. The Slit/Robo signaling pathway is also associated with early infantile epileptic encephalopathy [6] and autism [7]. While the Slits primarily signal through the Robo receptors, they have Robo-independent functions in the guidance of spinal commissural, cortical and olfactory axons [8], [9], [10]. This has led to the proposal that a new Slit receptor exists in mice, which acts either in conjunction or independently of Robos to regulate guidance.

Enhancer of ventral-axon guidance defects of *unc-40* mutants (Eva-1) was discovered in *C. elegans* [11]. It is required for the ventral guidance of the AVM axon, similar to the roles of the Slit/Robo orthologs, Slt-1/Sax-3 [11]. Further investigation revealed that Eva-1 co-localizes at the cellular membrane with Sax-3 and binds to Slt-1 [11]. Interestingly, ectopic expression of Eva-1 in cells with Slt independent Sax-3 functions confers Slt-1 dependence [11]. At present the expression pattern or functional role of the mammalian orthologue of Eva-1 remains unknown.

Down syndrome is the most common genetic cause of intellectual disability, resulting from trisomy of human chromosome 21. Recent research has highlighted the importance of the region 21q21-21q22.3 which is referred to as the Down syndrome critical region. Trisomy of this region results in the overexpression of ∼300 genes that are necessary, but not sufficient, for the pathologies present in Down syndrome [12]. Interestingly, the human orthologue of *Eva1c* (previously referred to as *Fam176C* or *C21orf63*) is located within the Down syndrome critical region (21q21-21q22.3). Understanding the expression and function of genes within this critical region may yield a better understanding of the etiology of the intellectual disability found in Down syndrome. We have characterized here the spatial and temporal expression patterns of EVA1C in regions of the developing mouse nervous system where the Slits display both Robo-independent and -dependent functions.

## Materials and Methods

### Animals

Protocols and use of animals in the described experiments were approved by the Animal Ethics Committee of the University of Queensland, which is registered as an institution that uses animals for scientific purposes under the Queensland Animal Care and Protection Act (2001). Embryos and post-natal wildtype C57BL/6 mice were examined.

### Immunofluorescence Staining

Embryos were decapitated and immersed in 4% paraformaldehyde for 24 hours at 4°C before cryopreservation in 30% sucrose in phosphate buffered saline (PBS) (pH 7.4). Post-natal mice were decapitated and their heads were immersed first, in 4% paraformaldehyde at 4°C for 24 hours, second, in 5% ethylenediaminetetraacetic acid (EDTA) at 4°C for 10 days, and finally, in 30% sucrose in PBS (pH 7.4) for cryopreservation. The tissue was coated with Tissue-Tek® OCT (Sakura Finetek, Tokyo, Japan) and snap frozen in isopentane cooled by liquid nitrogen. Cryostat sections (30 µm) of the head and spinal cord were mounted on Superfrost® Plus glass slides (Menzel-Gläser, Braunschweig, Germany) and stored at −20°C.

Cryostat sections were incubated in 2% Bovine serum albumin (BSA) in 0.1 M Tris buffered saline (TBS) with 0.3% Triton-X-100 for 1 hour. The sections were then incubated with the primary antibody in 2% BSA in 0.1 M TBS/0.3% Triton-X-100 for 15 hours at 4°C. The primary antibodies used in this study were rabbit anti-C21ORF63 (human EVA1C; 1∶200; gift of Takashi Angata, Riken Advanced Science Institute, Saitama, Japan) [13] and sheep anti-Rat-GAP43 (1∶400, Novus Biologicals, Colorado, USA). Sections were then washed with 0.1 M TBS/0.3% Triton-X-100 before been incubated with either donkey anti-rabbit Alexa Fluor® 594 (1∶200; Life Technologies, California, USA) or donkey anti-sheep Alexa Fluor® 488 (1∶200; Life Technologies, California, USA). Sections were rinsed in TBS and mounted in medium either with or without 4′,6-diamidino-2-phenylindole (DAPI) (Abcam, Cambridge, USA). Images were captured using an Olympus BX61 upright confocal microscope (Olympus Australia, Victoria, Australia) and adjusted for colour balance and brightness using Adobe Photoshop CS5 before being complied into figures using Adobe Illustrator CS6 (Adobe Systems Incorporated, California, USA). Controls sections incubated in the absence of primary antibody revealed only low background levels of red fluorescence (Fig. S1).

Four to six animals were examined at each time point from at least two different litters from independent mothers. Serial 30 µM cryostat sections were taken from the rostral tip of the olfactory bulb until the hippocampus at all ages. All sections were examined for the expression of EVA1C and GAP43. Similar staining patterns were observed in all animals at the same age.

### Western Blot

Tissue samples were extracted, placed in ice-cold 0.9% sodium chloride, in 0.1 M Tris (pH 7.4), 2 mM EDTA, 0.1 M phenylmethanesulfonylfluoride (PMSF), Aprotinin (500 KIU/ml) and Pepstatin A (1 µg/ml) and homogenized. The tissue extract was then centrifuged at 14,000 rpm for thirty minutes and the supernatant was collected and stored at −70°C. Membrane fractions were electrophoresed and immunoblotted as previously described [14].

## Results

### EVA1C is Expressed in Embryonic Spinal Cord

Spinal commissural neurons project axons across the floor plate into the contralateral ventrolateral funiculus in a process tightly controlled by Slit/Robo signaling [8], [15], [16]. Commissural axons express low levels of *Robo-1* and *-2* and high levels of *Robo-3.1* before midline crossing [8], [15], [16]. These axons respond to the chemorepulsive Slit ligands that are expressed at the ventral midline [15]. We examined here if EVA1C, the mammalian homologue of the novel *C. elegans* Slit-receptor Eva-1, is expressed in the mouse embryonic spinal cord at E10.5 (when commissural axons are pioneering the ventral commissural pathway) and at E17.5 (when the principal longitudinal axon tracts are established) using a previously generated polyclonal antibody raised against human EVA1C [12] (Fig. S2).

**Figure 1:**
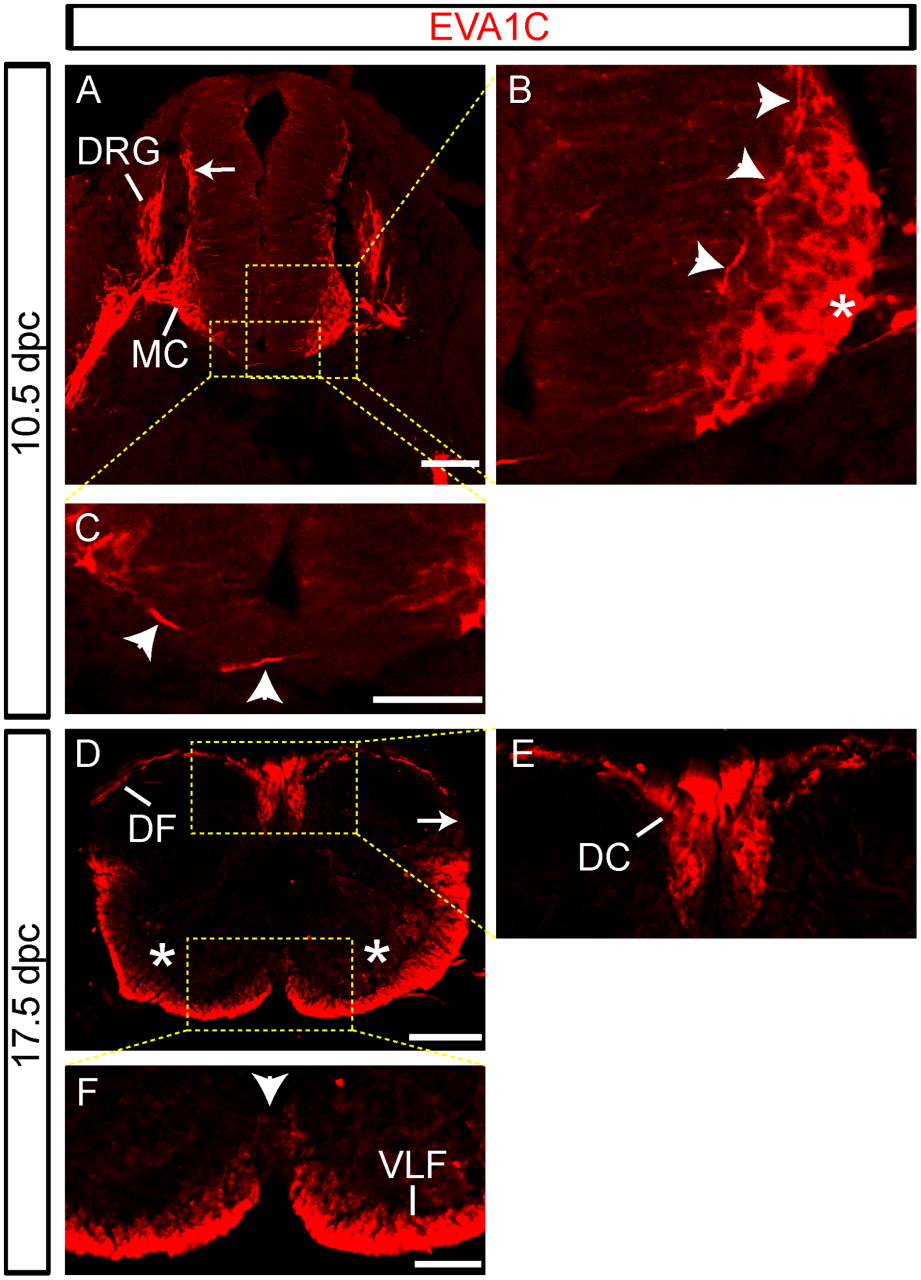
EVA1C expression in the developing spinal cord. (**A–F**) Transverse sections of the developing spinal cord at (**A–C**) 10.5 and (**D–F**) 17.5 dpc were immunostained for EVA1C and countersta. Boxed areas in A and D are represented in B–C and E–F, respectively. EVA1C was strongly expressed by the motor column (MC) and motor neurons (asterisks), and dorsal root ganglionic (DRG) neurons and their respective axons at (**A–C**) 10.5 dpc but not at (**D–F**) 17.5 dpc. EVA1C expression was detected in the dorsal root entry zone (arrows) at (**A**) 10.5 dpc, but not at (**D**) 17.5 dpc. (**A–C**) EVA1C expressing commissural axons can be observed coursing along the motor column and across the floor plate at 10.5 dpc (arrowheads). (**D–F**) EVA1C expression is lost in commissural axons as they cross the floor plate at 17.5 dpc. EVA1C is present in the ventrolateral funiculus (VLF), dorsal columns (DC) and dorsal funiculus (DF) at 17.5 dpc. Scale bar represents (**A**) 50 µm, (**B–C**) 25 µm, (**D**) 25 µm and (**E–F**) 10 µm.

At E10.5, EVA1C was expressed by motor neurons in the motor column and by their axons in the motor root as well by dorsal root ganglionic neurons and their central and peripheral axons (Fig. 1A). The EVA1C expressing sensory axons entered the dorsal horn at the dorsal root entry zone where they form the dorsal fasciculus [17], [18] (arrow, Fig. 1A). At higher magnification it was possible to observe a small number of commissural axons pioneering their trajectory from the dorsal spinal cord, along the inside border of the motor column and then across the midline floor plate [17], [19] (arrowheads, Fig. 1B–C). At E17.5, EVA1C was now clearly expressed in the ascending and descending longitudinal pathways that constitute the dorsal columns (DC, Fig. 1E), dorsal funiculus (DF, Fig. 1D) and ventrolateral funiculus (VLF, Fig. 1F). Interestingly, EVA1C was no longer expressed by the motor column (asterisks, Fig. 1D), spinal motor axons, dorsal root ganglion, or at the dorsal root entry zone (arrow, Fig. 1D) in these older embryos. EVA1C was also no longer expressed by the commissural axons as they coursed towards the midline and crossed the floor plate (arrowhead, Fig. 1F).

Together these results reveal that EVA1C is strongly and dynamically expressed in the spinal cord and peripheral nerve roots during embryogenesis. Notably, the expression of EVA1C by pre-crossing commissural axons is consistent with a role in regulating the function of the Slits during midline crossing. The expression of EVA1C in the major longitudinal tracts suggests possible roles of this receptor in regulating fasciculation and partitioning of axon subpopulations as they ascend and descend the cord.

### EVA1C is Present in the Developing Olfactory System

Olfactory sensory axons begin to extend from the primitive nasal cavity at E10, first contacting the presumptive olfactory bulb at E11.5 before spreading over its outer surface during late embryonic development. Most of these axons remain in the nerve fibre layer until the onset of glomeruligenesis at E15.5, when axons begin to enter into the olfactory bulb proper and establish glomeruli which become morphologically well-defined structures early in post-natal development [20]. *Robo2* is expressed by olfactory sensory axons that target the dorsomedial region of the olfactory bulb [21]. *Slit-1* and *Slit-2* are expressed by olfactory sensory axons that target the ventrolateral region of the olfactory bulb [9] while *Slit-1* and *Slit-3* are also expressed by bulbar cells in this ventrolateral region [21]. Together the Slits and Robos function to segregate olfactory sensory axons along the dorsoventral axis of the olfactory bulb [9], [21]–[23].

**Figure 2:**
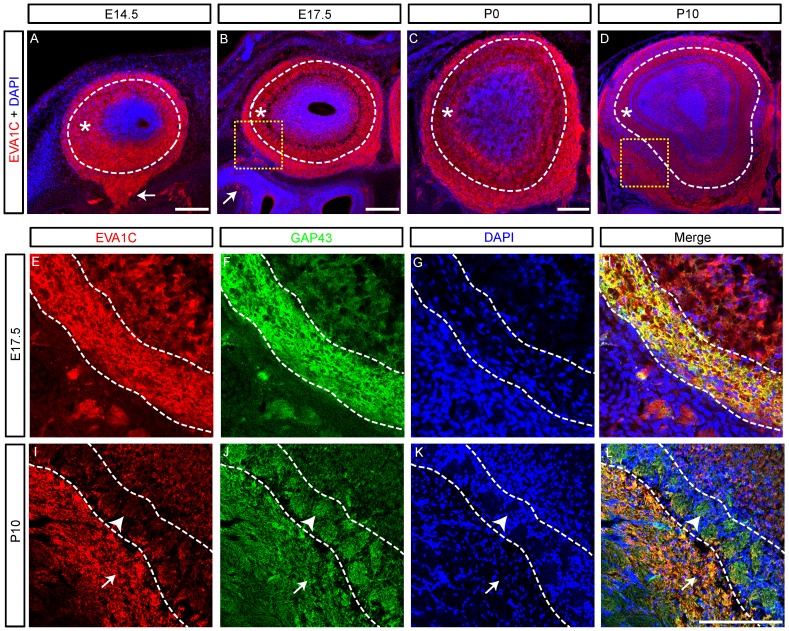
EVA1C is expressed in the olfactory bulb. (**A–L**) Coronal sections of the olfactory bulb at (**A**) E14.5, (**B, E–H**) E17.5, (**C**) P0 and (**D, I–L**) P10 were immunostained for (**A–D**) EVA1C and (**E–L**) EVA1C and GAP43. All sections were counterstained with DAPI. (**A–D**) EVA1C was strongly expressed in the olfactory nerve (arrows) and in the outer nerve fibre layer (demarcated by dashed line) of the developing and post-natal olfactory bulb. EVA1C was also expressed in the neuropil of the deeper layers of the olfactory bulb (asterisks) at all ages examined. Closer examination of the olfactory nerve layer (yellow boxed region in B and D) at (**E–H**) E17.5 and (**I–L**) P14 revealed that it was co-expressed with GAP43, a marker of olfactory sensory axons. (**I–L**) At P10, EVA1C was expressed at high levels in the olfactory nerve layer (arrows) but comparatively low levels in the glomerular layer (arrowheads). Scale Bars represent 100 µM. Dashed lines delineate: (**A–D**) the glomerular layer; (**E–H**) the olfactory nerve layer; (**I–L**) the glomerular layer.

We examined the expression of EVA1C in coronal sections of the olfactory bulb at E14.5, E17.5, P0 and P10 which encompasses the critical time window involving olfactory glomerular formation and maturation. At all ages EVA1C was strongly expressed throughout the outer layers of the olfactory bulb, which include the olfactory sensory axons (dashed lines demarcate nerve fibre and deeper layers, Fig. 2A–D) and the underlying external plexiform layer (asterisks, Fig. 2A–D). The olfactory sensory axons (arrows, Fig. 2A–B) arise from the olfactory neuroepithelium and project through the roof of the nasal cavity to reach the olfactory bulb. To confirm that EVA1C was expressed by olfactory sensory axons we co-stained sections at E17.5 for EVA1C and GAP43, a marker of these axons in the outer nerve fibre layer of the bulb [24]. In these double-stained sections it was clear that EVA1C was present on the growing olfactory sensory axons that encapsulated the bulb at this age (Fig. 2E–H). By P10, the olfactory sensory axons have formed distinct neuropil tufts called glomeruli that are surrounded by nuclei and lay beneath the nerve fibre layer of the olfactory bulb (arrowheads, Fig. 2I–L). Interestingly, EVA1C is exclusively expressed in the pre-terminal portions of olfactory sensory axons within both the nerve fibre layer (arrows, Fig. 2I–L) and as the axons enter the glomerular layer but it is not present in glomerular neuropil containing the terminal portions of the olfactory sensory axons (arrowheads, Fig. 2I–L). Punctate EVA1C staining continues to be present in the external plexiform layer deep to the glomerular layer at P10 (asterisk, Fig. 2D).

### EVA1C is Expressed in the Developing Visual System

**Figure 3:**
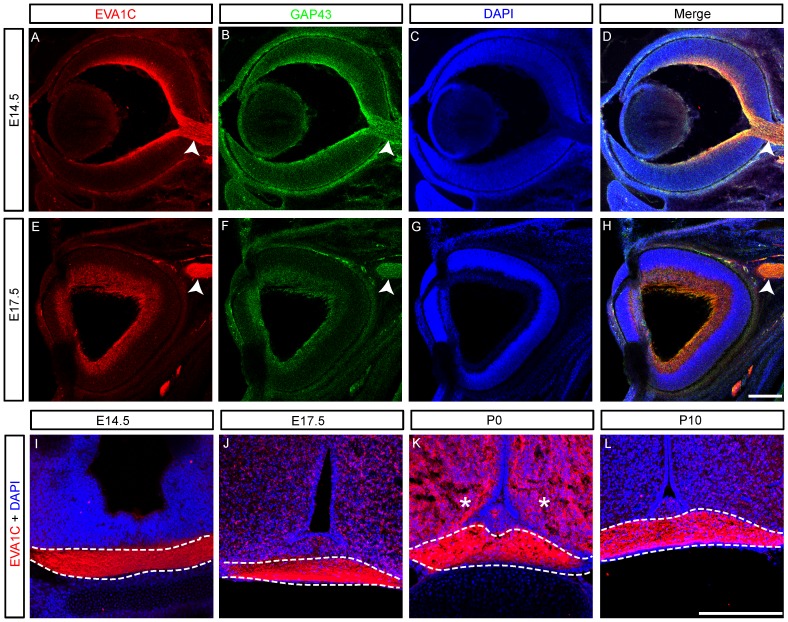
EVA1C is expressed by retinal cell ganglionic axons. Coronal sections of the developing (**A–H**) eye and (**I–L**) optic chiasm at (**A–D, I**) E14.5, (**E–H, J**) E17.5, (**K**) P0 and (**L**) P10 were immunostained for (**A–L**) EVA1C and (**A–H**) GAP43. All sections were counterstained with DAPI. EVA1C was strongly co-expressed with GAP43 by retinal cell ganglionic axons (**A–H**) as they exit the retina and form the optic nerve (arrowheads) and (**I–L**) as they cross the midline at the optic chiasm. (**K**) EVA1C expression was also expressed in the suprachiasmatic nucleus (asterisks) at P0. Scale bars represent 100 µM. Dashed lines demarcate the optic chiasm.

The majority of retinal ganglion cell (RGC) axons project from the retina and approach the commissural midline before entering into either the contralateral optic or ipsilateral optic tract between E14 and E17 [25]. The RGC axons that exit the retina during the later stages of development, between E17 and P0, almost exclusively extend into the contralateral optic tract [25]. Slit/Robo signalling plays an integral role in confining RCG axons within the optic chiasm [26]–[29]. We examined the expression of EVA1C in the visual system at E14.5, E17.5, P0 and P10 to assess its likely involvement in RCG axon guidance. EVA1C was highly expressed by GAP43-expressing RGC in the inner most layer of the retina and their axons which exit the rear of the eye to form the optic nerve between E14.5 (arrowheads, Fig. 3A–D) and E17.5 (arrowheads, Fig. 3E–H). Examination of coronal sections of the brain at the level of the optic chiasm revealed that EVA1C expression was maintained by RCG axons as they crossed the midline during development and early post-natal life (Fig. 3I–L).

Interestingly, in the suprachiasmatic nucleus, there was no expression of EVA1C at E14.5 (Fig. 3I), while at E17.5 there was weak mosaic staining in this nucleus (Fig. 3J). By P0 there was widespread staining throughout the nucleus (Fig. 3K), which then decreased to pre-E17.5 levels by P10 (Fig. 3L). Interestingly, there have been reports of transient expression of genes in the suprachiasmatic nucleus that have been associated with changes in circadian rhythms at birth [30]. Whether such mechanisms are driving this transient rise in EVA1C expression remains to be determined.

### EVA1C is Dynamically Expressed by Cortical, Hippocampal and Thalamic Axons

The corpus callosum is the largest axon tract in the mouse central nervous system and consists of cortical axons crossing from the ipsilateral to contralateral hemisphere. This commissural tract is pioneered by axons arising from the cingulate cortex at E16 [31], [32]. These axons form a discrete bundle that is located in the dorsal portion of the corpus callosum. However, not all cingulate axons cross the midline since some axons project ipsilaterally into the fornix [32]. Later growing axons arising from the neocortex then populate the ventral region of the corpus callosum [33]. While Slit/Robo signalling typically functions to restrict crossing axons to the corpus callosum, some Slit-mediated guidance of callosal axons is Robo-independent [3], [10], [34]–[36].

**Figure 4:**
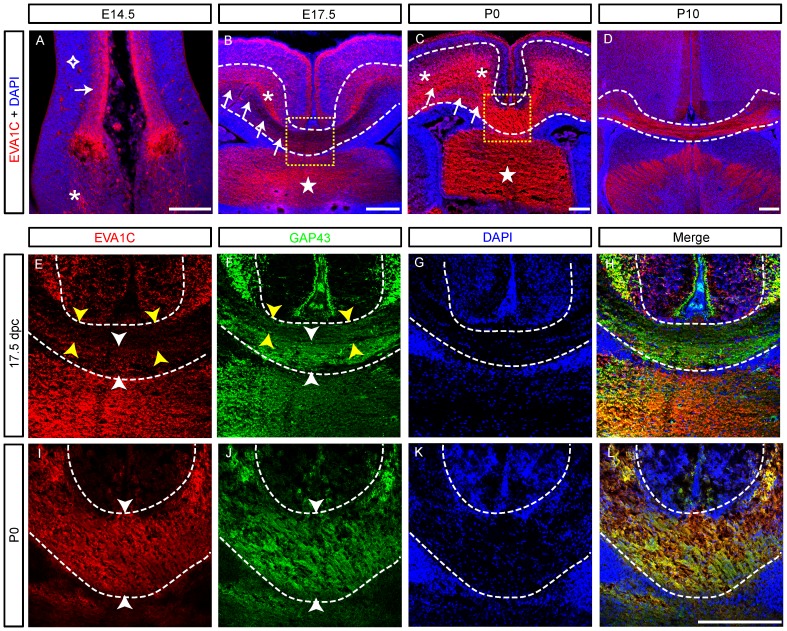
EVA1C expression in the corpus callsoum. Coronal sections of the developing cortex were immunostained for (**A–L**) EVA1C and (**E–L**) EVA1C and GAP43 at (**A**) E14.5, (**B, E–H**) E17.5, (**C, I–L**) P0 and (**D**) P10. All sections were counterstained with DAPI. (**A**) At E14.5, EVA1C was expressed in the cortical plate of the cingulate cortex (arrow) and in axons projecting into fornix (asterisk). EVA1C was not expressed in the underlying deeper layers of the cingulate cortex at this age (diamond). (**B**) At E17.5, axons arising from the cingulate cortex (asterisk) strongly express EVA1C. Axons form the neocortex (arrows) only weakly expressed EVA1C at this age (arrows). The yellow boxed area is presented in panels E–H. (**C**) At P0, EVA1C was expressed in the intermediate zone of the cingulate cortex and neocortex (asterisks). Axons entering the ventral corpus callosum (arrows) now express EVA1C. (**B, C**) EVA1C was highly expressed in the hippocampal commissure (stars). White dashed lines in panels B and C demarcate the intermediate zones and corpus callosum. (**D**) At P10, EVA1C was strongly expressed in the corpus callosum. (**E–H**) At E17.5, EVA1C was weakly expressed by callosal axons coursing diffusely (GAP43 staining) in the dorsal region of the corpus callosum (between yellow arrows). EVA1C was also weakly expressed by the more abundant GAP43 positive axons in the ventral corpus callosum (between white arrows). (**I–L**) At P0, EVA1C and GAP43 were uniformly co-expressed throughout the corpus callosum (between white arrowheads). Scale bars represent (**A**) 50 µM and (**B–L**) 100 µM. Dashed lines demarcate the corpus callosum.

We investigated EVA1C expression in coronal sections of the developing forebrain from E14.5 to P10, which incorporates the major period of formation and maturation of the corpus callosum. At E14.5, prior to the development of the corpus callosum, EVA1C expressing axons were observed arising from the cingulate cortex (arrow, Fig. 4A). At this age some of these axons had already begun to enter the presumptive fornix (asterisk, Fig. 4A). The deeper layers of the cingulate cortex, consisting of the intermediate zone and germinal layer [37], do not express EVA1C at E14.5 (diamond, Fig. 4A). By E17.5 the corpus callosum has formed and consists of axons from both the cingulate cortex, which cross in the dorsal region of the commissure, and the neocortex, which cross in the ventral portion of the commissure. At this age, EVA1C was strongly expressed in the intermediate zone underlying the cingulate cortex (asterisk, Fig. 4B) and more weakly expressed in the axons from the intermediate zone of the remaining neocortex (arrows, Fig. 4B). At the midline, the cingulate axons downregulate EVA1C and diffusely course in the dorsal corpus callosum (between yellow arrowheads, Fig. 4E–H). Neocortical axons continue to weakly express EVA1C as cross the midline in the ventral corpus callosum (between white arrowheads, Fig. 4E–H). In marked contrast, EVA1C is robustly expressed at E17.5 by commissural axons in the hippocampal commissure, which crosses ventral to the corpus callosum (star, Fig. 4B). At P0, EVA1C is now strongly expressed throughout the intermediate zone of the cingulate cortex and neocortex (asterisks, Fig. 4C). At this age, EVA1C was also strongly co-expressed with GAP43 by commissural axons through the dorsoventral depth of the corpus callosum (Fig. 4C; between white arrowheads, Fig. 4I–L). EVA1C continued to be expressed by axons in the hippocampal commissure at P0 (star, Fig. 4C). At P10, commissural axons in the corpus callosum maintain expression of EVA1C (dashed lines, Fig. 4D). Overall these results reveal that EVA1C is expressed by cortical and hippocampal axons, in a region overlapping with the expression of the Robos. Interestingly, the corpus callosal axons, particularly from the pioneer cingulate axons abundantly express EVA1C prior to crossing the midline. EVA1C appears to be restricted to the pre-crossing position of the embryonic commissural axons as the corpus callosum only weakly expresses this receptor (boxed area, Fig. 4B). However, by birth EVA1C becomes uniformly expressed by both the pre-crossing and crossing portions of these axons (boxed area, Fig. 4C). No such spatiotemporal restrictions in EVA1C localisation were observed on hippocampal commissural axons during this developmental period.

**Figure 5:**
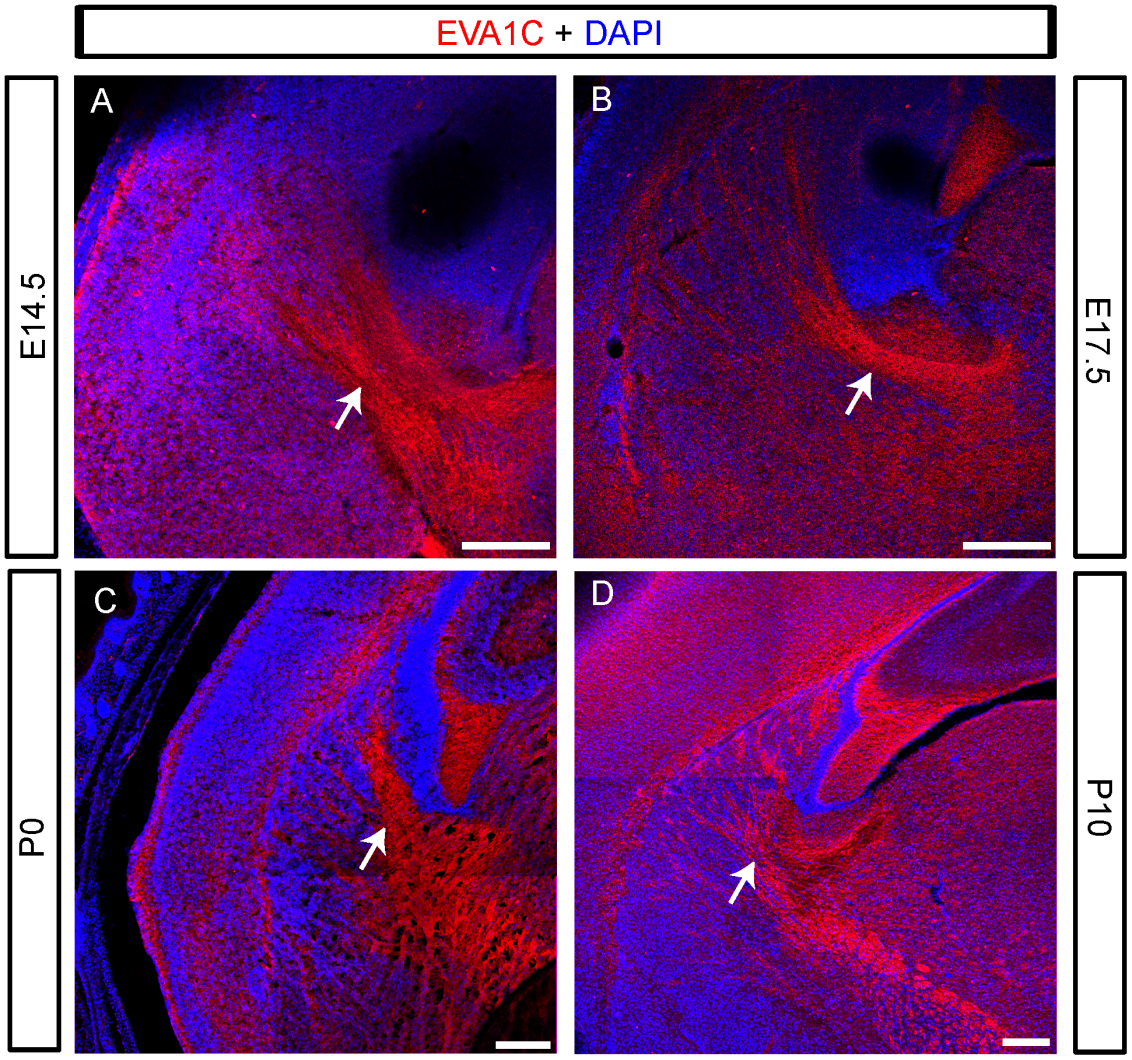
EVA1C is expressed by axons projecting through the internal capsule. Coronal sections of the forebrain at (**A**) E14.5, (**B**) E17.5, (**C**) P0 and (**D**) P10 were immunostained for EVA1C. All sections were counterstained with DAPI. (**A–D**) EVA1C was strongly expressed by axons in the internal capsule (arrows). Scale bars represent 100 µM.

The internal capsule is a large axon bundle that contains the reciprocal connections between the cortex and the thalamus. The capsule begins to form at E13.5, when thalamic axons enter the striatum [38]. By E15.5 these thalamic axons reach the lateral striatum and then serve as a guide for the cortical axons that then project through the striatum and reach the thalamus by E17.5 [38]. The formation of the developing internal capsule is known to be dependent on Slit/Robo signalling [3], [34]–[36]. We examined the expression of EVA1C in the internal capsule between E14.5 and P10. Unlike the commissural axons in the corpus callosum, we detected strong expression of EVA1C in the internal capsule throughout this temporal window (arrows, Fig. 5A–D).

## Discussion

Given the complexity of axon tract topologies in the mammalian brain there is a reasonable expectation that novel axon guidance molecules remain to be discovered. Support for this idea comes from the recent identification of candidate guidance receptors and ligands such as, axonal tyrosine phosphatase receptor PTPδ, the receptor for Slitrk3, a Slit and NTRK-like family member [39], Ig superfamily member and Shh receptor, brother of Cdo [40], neuronal transmembrane leukocyte common antigen-related phosphatase (LAR) [41], and leucine-rich transmembrane protein-3 (FLRT3), a ligand for Unc5D [42]. We have now shown here that EVA1C, the mammalian homologue of a novel Slit receptor in *C. elegans* is expressed in regions of the mouse nervous system known to involve Slit-mediated axon guidance. Since Slit is known to have Robo-independent functions, the expression of EVA1C in mouse suggests that this Slit receptor may participate in mammalian axon guidance. Our observations on the dynamic spatiotemporal expression patterns of this receptor suggests that its roles are highly context specific. For instance, in the spinal cord EVA1C is expressed by motor, sensory and commissural axons during early embryogenesis. Later, EVA1C is lost from these axons and it then begins to be expressed in the longitudinal tracts. Interestingly, EVA1C is restricted to the shafts and pre-terminal portions of olfactory sensory axons. EVA1C is absent from the terminal portions of these axons within the synaptic glomeruli. In the corpus callosum, EVA1C is selectively reduced on axonal shafts as they cross the midline during late embryogenesis. This exclusion from the commissural portion of the axons is transient since neonates exhibit uniform expression of EVA1C. Not all commissural axons display this spatiotemporal expression pattern since optic and hippocampal commissural axons ubiquitously express EVA1C as they cross the midline.

Spinal commissural neurons project axons across the floor plate into the contralateral ventrolateral funiculus in a process tightly controlled by Slit/Robo signaling [8], [15], [16]. Commissural axons express low levels of *Robo-1* and *-2* and high levels of *Robo-3.1* before crossing the floor plate at the ventral midline where the chemorepulsive Slit-1, -2 and -3 ligands are expressed [15], [16]. Robo-3.1 is believed to reduce Robo-1 and -2 levels in pre-crossing commissural axons and hence reduce the responsiveness of these axons to the midline chemorepulsive Slits. After reaching the midline, Robo3.1 is downregulated and then Robo-1, -2 and -3.2 are upregulated [15], [16]. This dynamic temporal regulation serves to facilitate the growth of commissural axons away from the floor plate. However, recent analyses of compound mutant mice reveal that additional Slit receptors must be expressed by commissural axons to account for their Slit-mediated repulsion from the midline [8]. In the absence of Robo-3 commissural axons fail to cross the midline, presumably due to the elevated expression of Robo-1/−2 on pre-crossing axons [8]. Since this phenotype is not fully rescued by the subsequent loss of Robo-1 and -2 it suggests that another Slit receptor is present on commissural axons. EVA1C expression on pioneer commissural axons is possibly responsible for this Robo-independent Slit repulsiveness. Since EVA1C is downregulated on commissural axons at E17.5, it can only be functionally active during early pioneering stages.

EVA1C is also expressed by spinal motor neurons and their axons at E10.5 but is lost by E17.5. Motor neurons have previously been shown to selectively express Robo1 at E10 while by E12.5 it is severely downregulated [43], [44]. However these studies did not provide evidence for Robo expression on the motor axons. It is not completely understood how motor axons are directed to grow out of the spinal cord. While an early study suggested that motor axons do not respond to Slit emanating from the floor plate [45] recent evidence indicates that Slit/Robo interactions are inhibiting DCC activity in motor axons and preventing these axons from growing towards Netrin-1 released by the floor plate [46]. Moreover a subset of motor axons called accessory motor axons have been shown recently to express Robo-2 which controls their Slit-dependent exit from the spinal cord [47]. The strong expression of EVA1C by motor neurons and their axons suggest that this Slit receptor could be responding to ventral cord derived Slit, in particular Slit-2 which is strongly expressed in the area encompassing the motor column [46]. EVA1C could conceivably be driving the exit of motor axons from the spinal cord and into the ventral root of the spinal nerve.

EVA1C is expressed within dorsal root ganglia, dorsal roots and at the dorsal root entry zone at E10.5 however this expression was lost by E17.5. The three Robo genes are also expressed by dorsal root ganglia and at the dorsal root entry zone, at least during early embryogenesis [43], [44], [48]. While Slit has not conclusively been demonstrated to be expressed in the dorsal root entry zone in mouse [8], [47], [49], analysis of Slit and Robo mutant mice revealed that some dorsal root sensory axons project aberrantly past the dorsal root entry zone towards the midline of the cord [50]. Because this phenotype is not fully penetrant in Robo-1 and Robo-2 double mutants it is possible that EVA1C may be acting redundantly to maintain the normal sensory axon trajectory. EVA1C is also expressed by axons in the dorsal funciculi, dorsal columns (which contains the corticospinal tract) and the ventrolateral funiculus in late stage embryos (Fig. 1). Both Robo-1 and -2 are believed to be expressed by corticospinal neurons and the corticospinal tract is distorted in Robo-2 mutant mice [3]. Despite some insight into the role of Slit/Robo interactions in the lateral positioning and sorting of axons in the caudal brain and ventral funiculus of the spinal cord [15], [51]–[53] further studies are needed in the spinal cord to better understand EVA1C function in relation to Slit/Robo interactions in these longitudinal tracts.

EVA1C is ubiquitously expressed by olfactory sensory axons, unlike Robo-2 and Slit-1 that have graded expression patterns in the olfactory system [21]. *Slit-1* and *Robo-2* cooperate to guide olfactory sensory axons to the dorsal region of the olfactory bulb [9], [21]–[23]. The defects present to the guidance of dorsal olfactory sensory axons in the absence of *Slit-1* or *Robo-2* are highly similar [21], indicating that if EVA1C is regulating the guidance of dorsal olfactory sensory axons it would do so in conjunction with Robo-2. However *Slit-1* is also required for the correct guidance of ventral olfactory sensory axons, independent of *Robo-2* function [9]. Since EVA1C is expressed by these ventral olfactory sensory axons it could be mediating the *Robo-2* independent functions of *Slit-1* in the olfactory system.

EVA1C was expressed by some of the major axon tracts in the forebrain, including the optic chiasm, the corpus callosum and the internal capsule. This expression was consistent with previous reports that Robo-1 and -2 are important for the development of major forebrain axon tracts [3], [34]. In particular, *Robo-1* and *-2* double mutants have a reduced corpus callosum, abnormal non-crossing bundles of callosal axons and aberrant ventral straying of callosal axons [3]. However this corpus callosum phenotype together with that described previously in *Robo-1* mutant mouse [35] is relatively mild in comparison to *Slit-2* single mutants [34] suggesting that other Slit receptors, such as EVA1C, are acting redundantly in the corpus callosum. We revealed that EVA1C was strongly expressed by retinal ganglionic axons along the optic nerve and across the optic chiasm, which is consistent with the role of Slit-2 in this region of the nervous system [26], [27], [54]. Since Slit-1 and -2 act to create a corridor to channel retinal axons across the midline, the absence of both of these Slits caused ectopic formation of a small second chiasm [27]. A similar phenotype was observed in mutant mice lacking Robo-2 [29], although it was not clear whether there was any quantitative difference between these animals that would support a role for EVA1C acting redundantly here. Finally we show that EVA1C is highly expressed by cortical and thalamic axons projecting through the internal capsule. Both the Slits and the Robos are required for dorsal turning of thalamic axons, enabling them to pass through the internal capsule [3], [34]. The loss of Slit/Robo signaling results in thalamic ectopically innervating the ventrally located hypothalamus [3], [34]. The expression of EVA1C within the early stages of formation of the internal capsule supports a role for EVA1C in the guidance of thalamic axons. However, due to phenotypic similarities between Slit and Robo mutants in this pathway it is likely that EVA1C is acting redundantly.

The nature of the interaction between EVA1C and Slit/Robo signaling in murine embryos remains unknown. In *C. elegans*, Eva-1 co-localizes with the Robo ortholog Sax-3 at the cellular membrane where it binds the Slit ortholog Slt-1 [11], [55]. Since Eva-1 was proposed to be required for all the Slt-1 dependent functions of Sax-3 it could regulate the responsiveness of Sax-3 expressing cells [11]. Given that EVA1C and Robos are not always co-expressed, it appears that EVA1C may also be mediating the Robo independent functions of the Slits. Heparan sulfate proteoglycans are important co-factors that regulate the interaction between Slit and Robo [56]. The binding of Eva-1 to Slt-1 in *C elegans* is promoted by 6-O-sulfation of Heparan Sulfate [55]. Heparan Sulfate 6-O-Sulfotransferase-1 (Hs6st-1) which modulates the 6-O sulfation of Heparan Sulfate is required for axon guidance processes at the optic chiasm and the corpus callosum in mice [57], [58]. Interestingly the loss of *Hs6st-1* mimics the loss of *Slit-2* at the optic chiasm, while the loss of both simultaneously results in a synergistic increase in the axon guidance defects [58]. These results have led to the proposal that *Hs6st-1* was modulating Slit signaling in the navigating growth cone [57]–[58]. Given the strong levels of EVA1C expression in retinal ganglion cell axons and the interaction between Eva-1, Slt-1 and 6-0-sulfaction of Heparan Sulfate in *C. elegans*, it is feasible that EVA1C is modulating Slit responsiveness in retinal ganglion cell axons in conjunction with *Hst6st-1*.

Taken together we have shown that the novel Slit receptor EVA1C is expressed in the developing mouse nervous system. The expression pattern of EVA1C lends support to the proposal that it is regulating Slit/Robo signaling either alone or in combination with the Robos, similar to its function in *C. elegans*. Given its presence in the Down syndrome critical region and the similarities in the defects present in Down Syndrome and Slit/Robo mutant embryos, it is tempting to speculate that *Eva1c* is associated with Down syndrome.

## Supporting Information

Figure S1 **Control staining of tissue sections.** Background non-specific levels of red fluorescence were observed when the primary antibody was omitted during immunohistochemical labeling of tissue sections. Similar background staining was observed at all ages and in all regions analysed. All sections were counterstained with DAPI.
(TIF)

Figure S2 **Western blot analysis of C21ORF63 antibody staining in mouse tissues.** Western blots of protein extracted from mouse brain, liver and heart in the presence and absence of the anti-EVA1C (C21ORF63) antibody. No bands were identified when no primary antibody was used. The C21ORF63 antibody specifically labeled a 51 kDa band at the predicted size of EVA1C. A non-specific low molecular band was present at ∼27 kDa in all extracts. As previously shown, this band is undetected by immunofluorescence staining of cells [13]. Only cells transfected with EVA1C (C21ORF63) were positively stained by this antibody [13].
(TIF)
