# Supplementary figures and images for: The Expression Pattern of EVA1C, a Novel Slit Receptor, Is Consistent with an Axon Guidance Role in the Mouse Nervous System
Source: PLoS One. 2013 Sep 9;8(9):e74115. doi: 10.1371/journal.pone.0074115 (PMC3767613; doi:10.1371/journal.pone.0074115)

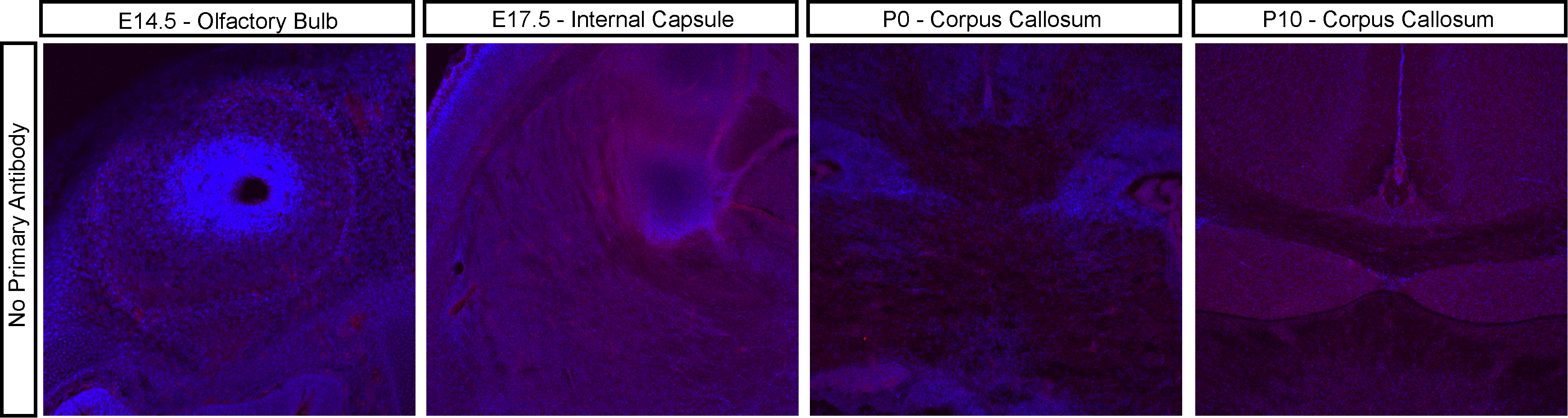

Supplement: Figure S1 — Control staining of tissue sections. Background non-specific levels of red fluorescence were observed when the primary antibody was omitted during immunohistochemical labeling of tissue sections. Similar background staining was observed at all ages and in all regions analysed. All sections were counterstained with DAPI. (TIF) [file pone.0074115.s001.tif]

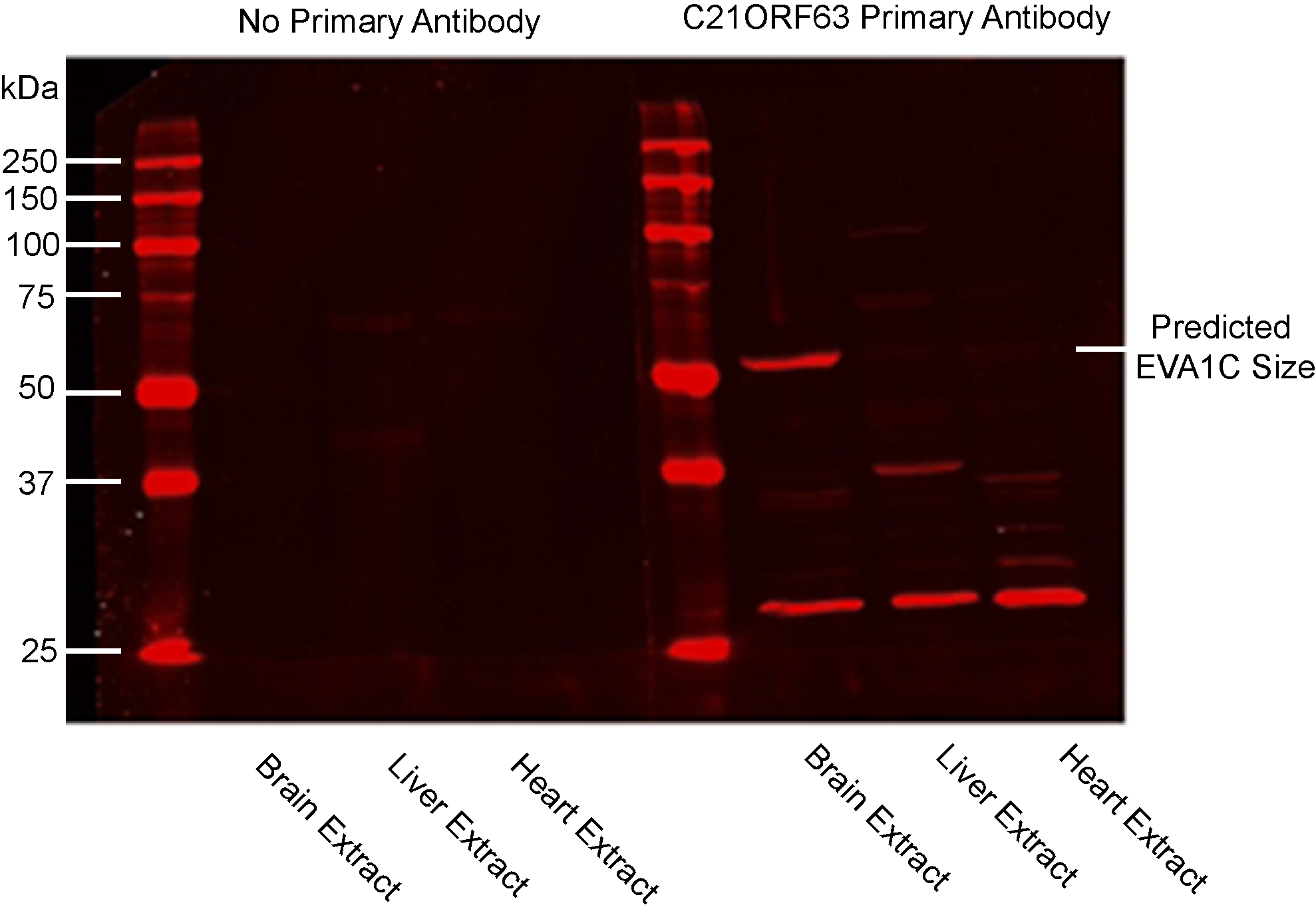

Supplement: Figure S2 — Western blot analysis of C21ORF63 antibody staining in mouse tissues. Western blots of protein extracted from mouse brain, liver and heart in the presence and absence of the anti-EVA1C (C21ORF63) antibody. No bands were identified when no primary antibody was used. The C21ORF63 antibody specifically labeled a 51 kDa band at the predicted size of EVA1C. A non-specific low molecular band was present at ∼27 kDa in all extracts. As previously shown, this band is undetected by immunofluorescence staining of cells [13]. Only cells transfected with EVA1C (C21ORF63) were positively stained by this antibody [13]. (TIF) [file pone.0074115.s002.tif]
